# An optimistic protein assembly from sequence reads salvaged an uncharacterized segment of mouse picobirnavirus

**DOI:** 10.1038/srep40447

**Published:** 2017-01-10

**Authors:** Gabriel Gonzalez, Michihito Sasaki, Lucy Burkitt-Gray, Tomonori Kamiya, Noriko M. Tsuji, Hirofumi Sawa, Kimihito Ito

**Affiliations:** 1 Bioinformatics Division, Research Center for Zoonosis Control, Hokkaido University, Sapporo, Japan; 2 Department of Molecular Pathobiology, Research Center for Zoonosis Control, Hokkaido University, Sapporo, Japan; 3 UCD Conway Institute of Biomolecular and Biomedical Science, University College Dublin, Dublin, Ireland; 4 Immune Homeostasis Laboratory, Biomedical Research Institute, National Institute of Advanced Industrial Science and Technology, Tsukuba, Japan; 5 Global Institution for Collaborative Research and Education, Hokkaido University, Sapporo, Japan; 6 Global Virus Network, Baltimore, MD, USA

## Abstract

Advances in Next Generation Sequencing technologies have enabled the generation of millions of sequences from microorganisms. However, distinguishing the sequence of a novel species from sequencing errors remains a technical challenge when the novel species is highly divergent from the closest known species. To solve such a problem, we developed a new method called Optimistic Protein Assembly from Reads (OPAR). This method is based on the assumption that protein sequences could be more conserved than the nucleotide sequences encoding them. By taking advantage of metagenomics, bioinformatics and conventional Sanger sequencing, our method successfully identified all coding regions of the mouse picobirnavirus for the first time. The salvaged sequences indicated that segment 1 of this virus was more divergent from its homologues in other *Picobirnaviridae* species than segment 2. For this reason, only segment 2 of mouse picobirnavirus has been detected in previous studies. OPAR web tool is available at http://bioinformatics.czc.hokudai.ac.jp/opar/.

Advances in Next Generation Sequencing (NGS) technologies allowing the generation of large numbers of nucleotide reads from low-volume samples have made microbiome analyses using environmental, fecal, and other samples possible. Analysis of these samples may indicate the presence of novel microorganisms. When these microorganisms are genetically similar to known species, the sequences can be identified by comparison against the genomes of characterized species or the lowest common ancestor for those sequences^1^,^2^.

Although a metagenomics approach for identification of new species from NGS products is powerful, it can prove difficult when the sequence of interest is highly divergent from the closest genomic references. We faced such a situation when we detected part of a picobirnavirus genome whilst analyzing the virome isolated from fecal samples of laboratory mice.

Picobirnavirus (PBV) was first isolated from human fecal samples in 1988. It is characterized as a two-segmented double-stranded RNA virus^3^ with a small non-enveloped capsid (35–41 nm in diameter). Segment 1 (2.2–2.7 kb) encodes a non-structural protein (S1NSP) and the capsid protein (S1CP). Segment 2 (1.2–1.9 kb) encodes the RNA-dependent RNA-polymerase^4^,^5^ (S2RDRP). PBVs have been isolated in fecal samples from a variety of species including humans, pigs, cattle, dogs, monkeys, rabbits, rats, cats, snakes and birds^3^,^6^,^7^,^8^,^9^. The wide host range, despite the small and simple genome, highlights the adaptive potential of PBVs. Infections in humans have been associated with gastroenteritis, diarrhea and opportunistic infections in immunocompromised patients, in addition to occasional asymptomatic infections^4^,^5^,^7^. Phylogenetic comparison of multiple PBV strains from humans and other species have indicated potential zoonotic transmission^4^.

There is a large difference in the number of publicly available sequences for segment 1 and segment 2 of PBV. There are 933 nucleotide sequences from PBV in the National Center for Biotechnology Information (NCBI) database as of April 2016, with 48 corresponding to segment 1 and 885 to segment 2 of the genome. Segment 2, which encodes S2RDRP, is sufficiently conserved to allow the design of sequencing primers even for novel PBV species. However, segment 1is highly divergent, especially the region encoding S1NSP, which presents a technical challenge to the further genetic characterization of PBV species^10^. The low number of publicly available complete PBV genome sequences also hinders the understanding of the epidemiology and evolution of the *Picobirnaviridae* family^5^.

In this paper, we describe a new bioinformatics method with which we salvaged nucleotide sequences of a novel mouse picobirnavirus from NGS products. A key premise of this method is collecting full nucleotide information from sequence reads which partially align to reference amino acid sequences. This method has been named Optimistic Protein Assembly from Reads (OPAR). It is publically available online at http://bioinformatics.czc.hokudai.ac.jp/opar/. We anticipate that OPAR web tool will contribute to the discovery and characterization of new viral species.

## Results

### Detection of sequence reads from segment 2 of picobirnavirus in mouse virome

Fecal samples obtained from 12-week-old BALB/c mice were sequenced using the Illumina MiSeq NGS platform. DIAMOND^11^ was used to align NGS reads against known proteins in the database of non-redundant protein sequences (NR/NT^12^) of the NCBI. In total 846,454 NGS reads were aligned to known protein sequences in NR/NT, with 237,830 aligned to viral proteins. Of these, 234,730, 1,638 and 846 were mapped onto proteins from PBV, Tobamovirus and Flavivirus, respectively. In excess of 98% of reads mapping to viral proteins were associated with PBV. These PBV-associated reads mapped onto multiple PBV species, including mouse PBV, dromedary PBV and human PBV, suggesting that the reads might originate from an uncharacterized PBV.

136,893 of the 234,730 identified PBV reads aligned to S2RDRP of PBV/mouse/USA/2008^7^. *De novo* assembly of these reads produced a single sequence of 1,110 nt which was identified using BLASTN^13^ as segment 2 of PBV/mouse/USA/2008 (JF755419.1), with 92% identity and 78% query coverage.

We have designated this novel PBV strain mouse picobirnavirus strain Japan 2015 (PBV/mouse/JPN/2015).

### Deduced nucleotide sequences by Optimistic Protein Assembly from Reads (OPAR)

A high coverage was found for segment 2 of PBV/mouse/JPN/2015 in the sequence reads obtained from NGS analysis. However, no reads could be assigned to segment 1 of PBV/mouse/JPN/2015 using BLASTN. This indicated that segment 1 of PBV/mouse/JPN/2015 was highly divergent from other known PBV species.

**Figure 1:**
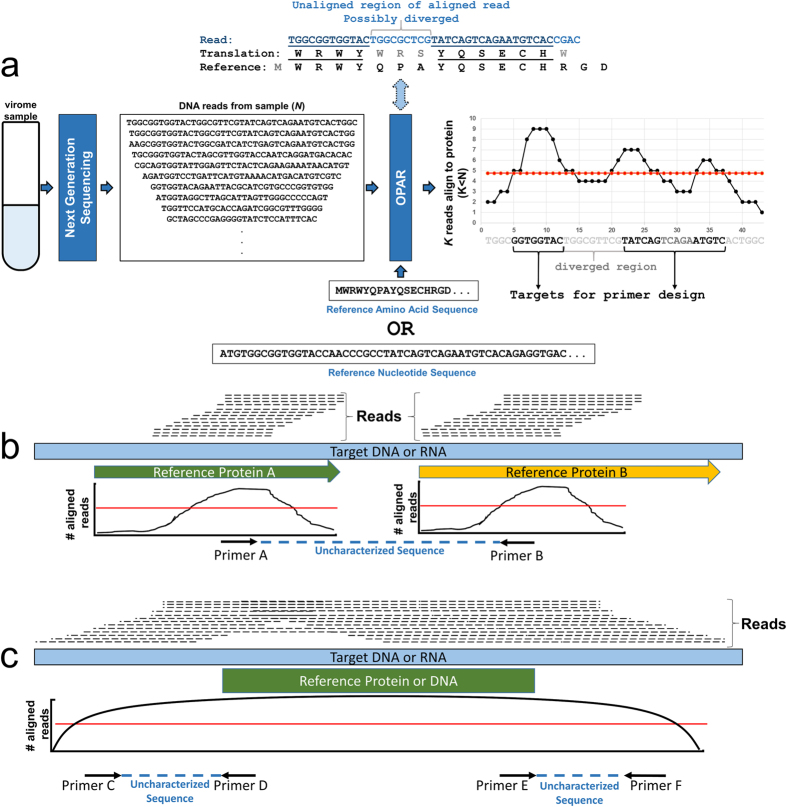
Diagram of OPAR approach to deduce nucleotide sequences. (**a)** Flowchart of the steps for aligning *N* reads from a virome to a specific amino acid or nucleotide sequence used as a reference. Aligned and unaligned segments of the *K* aligned reads are the input for building a consensus sequence used for designing primers. (**b)** Example OPAR usage for designing primers from consensus sequences on proteins. Primers A and B designed from consensus sequences respectively located in proteins A and B allow us to analyze the nucleotide sequence between these regions. (**c)** Example OPAR usage for designing primers from consensus sequences located outside of characterized sequences. Primers C and F designed from consensus sequences respectively located upstream and downstream of an already characterized sequence allow us to analyze the uncharacterized novel sequences. Primers D and E are designed on the characterized section of the sequence.

Proteins in segment 1 of human picobirnavirus were used as references to construct two potential contigs corresponding to partial nucleotide sequences for S1NSP and S1CP (Fig. 1a,b) through the OPAR method. Human picobirnavirus^14^ (NC_007026) S1NSP and S1CP were used as references since no sequences for segment 1 of PBV/mouse/USA/2008 are publicly available. In total 13,827 and 147,968 of viral reads aligned to the S1NSP and S1CP protein sequences respectively. Although both proteins are located on the same viral segment, ten times more reads mapped to the S1NSP than the S1CP. This is suggestive of less conservation in the S1NSP between mouse PBV and human PBV, compared to the S1CP.

**Figure 2:**
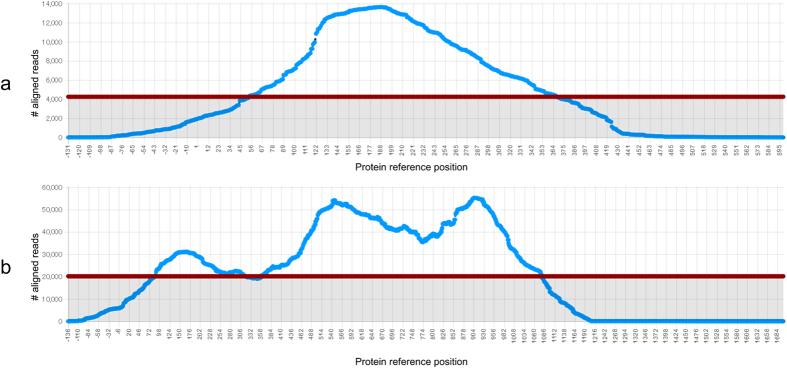
Coverage of reads aligned to PBV/mouse/JPN/2015 segment 1 proteins outputted by OPAR. The ordinate axes represent the count of reads aligned to each particular section of the protein sequence. The abscissae represent the relative nucleotide position inferred from the aligned amino acid sequences of the respective proteins. Horizontal red lines represent the threshold set to the minimum expected average with 90% confidence, 4254 and 20,167 reads in (**a**) and (**b**), respectively. The results correspond to reads aligned to homologues in human picobirnavirus of proteins in segment 1 (**a)** non-structural protein and (**b)** capsid protein.

These assembled nucleotide sequences encoding S1NSP and S1CP (Fig. 2a,b) allowed us to design primers No. 1 and No. 2, located in the coding region of the S1NSP and in the S1CP respectively (Table 1). A partial sequence of 1,095 bps from segment 1 of PBV/mouse/JPN/2015 was obtained as the PCR product. This was then used as a reference for the OPAR method with non-aligning regions of the aligned reads collected to extend the sequence (Fig. 1c). A second set of primers, Nos 3 and 4 (Table 1), were designed and this process iterated a further three times, with a total of seven primers produced for segment 1 (Table 1). The final sequence was amplified using the RACE protocol to complete the 3′-terminal section of segment 1. This process was repeated using primers No. 8 and No. 9 (Table 1) in segment 2 of PBV/mouse/JPN/2015 to complete the region encoding the S2RDP. The complete sequences for coding regions in segment 1 and 2 are publicly available with accession numbers LC110352 and LC110353, respectively.

We confirmed our sequences through the standard BLASTN method. In total, 494,436 of the original NGS reads mapped to segment 1 and 199,906 to segment 2 of PBV/mouse/JPN/2015 when LC110352 and LC110353 were provided as references.

### Genetic characterization of PBV/mouse/JPN/2015 genome

We identified a near-complete genome for PBV/mouse/JPN/2015, with a 2,490 base pair sequence isolated from segment 1 and a 1,409 base pair sequence from segment 2. Only the 3′ terminus of segment 1 could be amplified using the RACE protocol; neither terminus of segment 2 could be isolated. However, start and end codons for open reading frames (ORFs) were detected for all PBV proteins, indicating that the complete coding regions of PBV/mouse/JPN/2015 have been identified.

The isolated PBV segment 1 has a G + C content of 40.12%. It contains two ORFs predicted to encode S1NSP and S1CP. The S1NSP protein is 241 amino acids in length, encoded by nucleotides at positions 5 to 730 on the determined segment 1 sequence. Although the function of this protein remains to be confirmed, we ascertained the presence of eight repetitions of the motif ExxRxNxxxE which has been reported as a common factor in S1NSP proteins across PBV species^10^. The second segment 1 protein, S1CP, which is assembled into the capsid^15^, is 577 amino acids long and encoded at positions 727 to 2460 on the identified nucleotide sequence. The ORFs of S1NSP and S1CP overlap at four positions from 727 to 730. The third nucleotide (A) of the final codon and the opal termination codon (UGA) of S1NSP overlap with the initiation codon (AUG) and the first nucleotide (A) of the second codon of S1CP. A frameshifting of −1 at this overlapping region has previously been proposed^14^. Pairwise amino acid comparison of PBV/mouse/JPN/2015 proteins to the 12 available complete protein sequences of S1NSP and 16 of S1CP from other PBV species showed low amino acid identities for both segment 1 proteins (Table 2).

The sequenced region of segment 2 has a G + C content of 47.76%. It encodes the 402 amino acid-long S2RDRP protein at position 173 to 1381 on the identified nucleotide sequence. The S2RDRP ORF encodes the RdRp, which is responsible for the replication of double-stranded RNA segments of PBV^6^. Three motifs, DxT/SxxD, SGxxxT and GDD, are known to be conserved in the RdRp protein of PBV species and other dsRNA viruses^4^,^6^. These motifs are present as DFTKFD, SGSGGT and GDD in PBV/mouse/JPN/2015.

**Figure 3:**
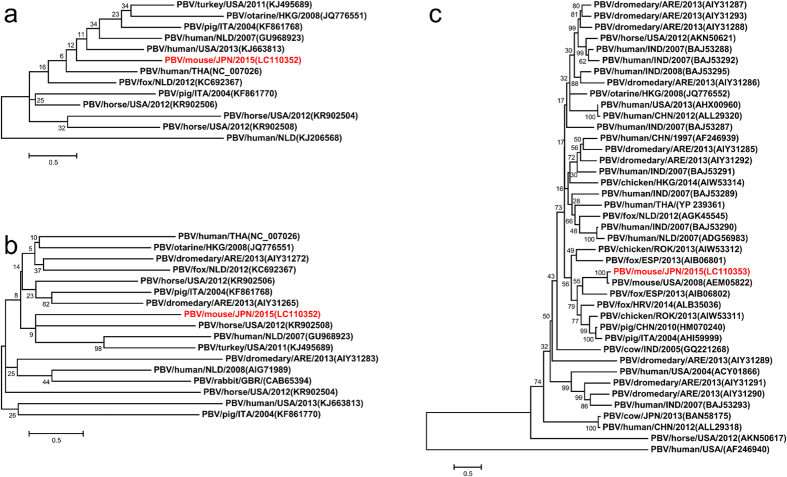
Phylogenetic trees of putative PBV proteins. The percentage of trees inferred by Maximum Likelihood in which the associated taxa clustered together are shown next to the branches. Accession numbers of sequences are displayed between parentheses. The taxa of novel characterized PBV/mouse/JPN/2015 proteins are written in red. The trees correspond to (**a)** segment 1 non-structural protein, (**b)** segment 1 capsid protein and (**c)** segment 2 RNA-dependent RNA polymerase.

Phylogenetic analysis of PBV/mouse/JPN/2015 proteins and homologs in other species indicated that PBV/mouse/JPN/2015 is highly divergent from other PBVs (Fig. 3a–c). In particular, trees of S1NSP (Fig. 3a) and S1CP (Fig. 3b) showed longer branches than those of S2RDRP (Fig. 3c), suggestive of higher divergence in segment 1 than segment 2.

## Discussion

The first rodent PBV was identified as double-stranded RNA virus with a bi-segmented genome, isolated from the intestinal contents of wild black-footed pygmy rice rats (*Oryzomys nigripes* or *Oligoryzomys nigripes*) in Brazil^3^. Two further partial genome sequences of segment 2 from rodent-borne PBVs have been reported^7^,^9^. However, segment 1 of rodent-borne PBV has not yet been sequenced. In this study we identified a new strain of mouse picobirnavirus in fecal samples from laboratory mice through a metagenomics approach. We named the new strain PBV/mouse/JPN/2015 and identified the full coding sequence for each segment. Segment 2 of PBV/mouse/JPN/2015 was detected in fecal samples using metagenomics analysis. OPAR was used to assemble partial nucleotide sequences for segment 1 of PBV/mouse/JPN/2015, which could not be identified using a conventional BLASTN approach. Using the partial assembly from OPAR, we designed primers and successfully detected the full sequence for coding regions in both segments with Sanger sequencing.

The proteins encoded in segment 1 and segment 2 harbored conserved amino acid motifs characteristic of PBV. Average evolutionary distances from other PBV species at the amino acid level are 1.80 ± 0.50, 2.18 ± 0.23 and 1.27 ± 0.90 for S1NSP, S1CP and S2RDRP respectively (Fig. 3a–c), under a JTT + G substitution model. The S1CP protein encoded on segment 1 has diverged faster than the segment 2 protein (S2RDRP). These distances highlight how divergent this virus is from other known PBV species. The greater evolutionary distances explain why fewer nucleotide reads could be aligned to segment 1 than segment 2. These results characterize PBV/mouse/JPN/2015 as a member of PBV with a highly divergent segment 1 genome.

We identified full sequences for coding regions of PBV/mouse/JPN/2015 from laboratory mice in Japan. PBV/mouse/USA/2008 — a rodent-borne PBV isolated from wild house mice (*Mus musculus*) in the USA^7^ — had 92% nucleotide identity to PBV/mouse/JPN/2015 in segment 2. Segment 2 of PBV/mouse/JPN/2015 also showed 94% nucleotide identity to a partial PBV sequence isolated from *Rattus norvegicus* in Brazil (unpublished, accession no. GU230581). This suggests PBV/mouse/JPN/2015 and closely related PBV strains may be widely distributed in rodent species. The PBV/mouse/JPN/2015 genome will provide a reference sequence for detection of further rodent-borne PBVs.

PBV/mouse/JPN/2015-positive mice were reared in a specific-pathogen-free animal room and did not display any signs of infection such as gastroenteritis or diarrhea. Similarly, PBVs have been detected in captive Wistar rats (*Rattus norvegicus*) without diarrhea^9^. This suggests that PBV may cause asymptomatic infection in rodents. Further studies on PBV including using *in vitro* cell cultures are needed to characterize this infection and its epidemiology in rodents, including laboratory animals.

OPAR is based on the assumption that the nucleotide sequences of genes encoding homologous proteins show faster phylogenetic change than the amino acid sequences. A number of reports have suggested that some regions in viral genes naturally undergo purifying selection^16^,^17^. As amino acid sequences are more stable than their encoding nucleotide sequences, amino acid sequences can provide a scaffold for aligning multiple nucleotide reads to amino acid motifs under negative selection. Once nucleotide reads have been aligned to a reference protein, the non-aligning regions of these reads are used to deduce a possible consensus for the original coding sequence. The aligning regions are used to establish the position of the read within the protein, while the unaligned parts are assumed to be divergent parts of the sequence. Low nucleotide diversity at sites in the unaligned sections strengthens the reliability of the assembled consensus sequence, which is returned with a confidence level directly proportional to the count of aligned reads.

There are several NGS pipelines currently available online^18^. Most online pipelines use reference mapping algorithms such as Bowtie^19^ and BWA^20^, which are designed to build a consensus sequence from aligned reads. These approaches are efficient when the target sequence is close to the provided reference. Although we could detect sequences homologous to segment 2 of PBV using reference mapping and simple BLASTN searches, we were unable to identify sequences from segment 1 using these approaches. Although MEGAN found clear taxonomical evidence of mouse PBV in the sample, a *de novo* assembly of the sequences assigned as PBV did not produce a satisfactory result. The OPAR method gathers sequence information from unaligned sections of reads locally aligned to proteins. Using this approach, we successfully designed primers which enabled almost complete sequencing of segment 1 of mouse PBV for the first time. To the best of our knowledge, there are no other online pipelines which have implemented this approach.

OPAR is designed to be simple and straightforward for all potential users, and does not require any knowledge of the underlying alignment tools. The main purpose of this method is to salvage reads from metagenomics datasets which could be distantly related to another characterized species. The only required input is a set of reads or contigs from a next generation sequencing process, in either FASTA or FASTQ format. A specific protein or nucleotide sequence can also be provided to be used as reference when aligning the reads. To improve the performance of OPAR when used to query a set of reads against all known proteins in NR/NT, the alignment is performed by a local instance of DIAMOND against a local copy of the NCBI NR database^12^, updated weekly. Currently, local alignments using BLAST can also be performed; BLASTX^13^ or BLASTN can be used to align against a provided amino acid or nucleotide sequence for a specific protein.

In conclusion, we have produced the first sequence for segment 1 of a mouse picobirnavirus species, determined with a metagenomics and bioinformatics method. This strain was found in fecal samples of laboratory mice and named PBV/mouse/JPN/2015. Although much of the epidemiology of PBV remains to be characterized, our study suggested that PBV/mouse/JPN/2015 and its close relatives may be widely distributed throughout rodent species. We have also demonstrated how assembly guided by amino acid identities can be used to salvage information from nucleotide sequences that are highly divergent from the closest references. The pipeline of bioinformatics analyses named OPAR is available online at http://bioinformatics.czc.hokudai.ac.jp/opar/.

## Materials and Methods

### Mouse virome sequencing and metagenomics analysis

Six-week-old female BALB/c mice purchased from Sankyo-Lab (Tsukuba, Japan) were housed under specific pathogen-free conditions at the National Institute of Advanced Industrial Science and Technology (AIST; Tsukuba, Japan). Fecal samples were obtained from five mice at 12 weeks of age and stored at −80 °C until analysis. For viral nucleic acid extraction and double-stranded cDNA synthesis from fecal samples, we followed the method of Sasaki *et al*.^21^. The isolated double-stranded cDNA was used for metagenomics library preparation with the Nextera XT DNA sample preparation kit (Illumina, San Diego, CA). Sequencing was performed on the Illumina MiSeq platform (Illumina). The reads from the mouse (*Mus musculus*) virome were compared against NCBI NT/NR database by using the DIAMOND, BLASTN and BLASTX programs with an expected value (e-value) ≤10^−4^. The results were then classified and summarized using MEGAN^1^ with LCA minimum score, maximum expected and minimum support set to 25, 0.01 and 5, respectively. *De novo* assemblies were performed using CLC Genomics Workbench 8.5.1 (CLC bio, Aarhus, Denmark).

All experiments involving animals were performed in accordance with the ethical guidelines of the National Institute of Advanced Industrial Science and Technology (AIST). The protocols were approved by the Animal Welfare Committee of AIST.

### Optimistic Protein Assembly from Reads (OPAR) method

We developed a novel method, Optimistic Protein Assembly from Reads (OPAR), which salvages viral sequence reads that are highly divergent from the reference sequence. The OPAR method consists of an alignment step followed by a merging step. In the alignment step, either BLASTX^13^ or DIAMOND^11^ is used to align nucleotide sequences to protein references. For this study, we used an e-value of 10^−4^ for BLASTX and DIAMOND to reduce the false positive rate. In the merging step, OPAR constructs consensus sequences by combining the aligned and unaligned sections of these aligned reads, assuming that non-aligning regions are divergent segments of the proteins (Fig. 1a).

The output of the chosen alignment tool, e.g. BLASTX, is mapped onto the reference protein using multiple sequence alignment to determine the positions of the aligned segments. In contrast to other methods, OPAR maintains the unaligned sections of the sequences (Fig. 1b). Once all aligning reads have been mapped through multiple sequence alignment, their sequences are merged into a consensus sequence using the most frequently occurring nucleotide for each position.

In summary, OPAR determines aligning regions using local alignments tools (DIAMOND, BLAST or BLASTX). It then collects the entire nucleotide sequences of reads with any aligning segments, which are then used to construct a consensus sequence.

### Sequencing of mouse picobirnavirus segments

To confirm the consensus sequences, deduced by OPAR by aligning reads from the sequenced virome to human picobirnavirus proteins S1NSP (YP_239359.1) and S1CP (YP_239360.1) at an e-value ≤10^−4^, we designed primers to connect the consensus sequences in each protein, thus completing segment 1. This process was repeated to complete segment 2, which encodes the S2RDRP. Conventional RT-PCR was performed using Tks Gflex DNA polymerase (Takara Bio, Ohtsu, Japan) and the primers designed with PrimerBlast^22^ (Table 1). The PCR amplicons were sequenced by the Sanger method using the BigDye Terminator v3.1 Cycle Sequencing kit (Life Technologies, Carlsbad, CA). To sequence the terminal regions, a Rapid Amplification of cDNA Ends (RACE) protocol was followed using the SMARTer RACE cDNA Amplification Kit (Takara Bio).

### Phylogenetic analyses

Homologous protein sequences for S1NSP, S1CP and S2RDRP were obtained from the NCBI NR/NT database by querying each predicted amino acid sequences using BLASTP^13^. Short partial sequences were omitted, and only complete sequences or those seemingly near completion (>80%) were used for further analyses. Multiple sequence alignments were built with MAFFT^23^ using the FFT-NS-I algorithm, setting the scoring matrix to JTT200 and allowing for gappy regions, with other parameters kept at the default values. Identity percentages were estimated on BioEdit^24^. The phylogenetic trees and evolutionary distances were inferred using only amino acid sites with higher than 80% coverage in MEGA6^25^. 105 out of 241, 448 out of 577 and 402 out of 402 amino acid sites were used to infer the phylogenetic trees for S1NSP, S1CP and S2RDRP, respectively. Phylogenetic pairwise evolutionary distances were estimated using the JTT matrix-based model^26^ considering the rate variation among sites modeled with a gamma distribution (JTT + G) with parameters 2.76, 1.54 and 0.64 for S1NSP, S1CP and S2RDRP, respectively, estimated with MEGA6. Phylogenetic trees were inferred using a Maximum Likelihood with Le-Gascuel (LG)^27^ substitution model, and a gamma distribution estimated with five discrete categories to model different rates among sites (+G). The phylogeny of each tree was tested using a bootstrap method with 1000 pseudo-repetitions. For readability, names of PBV sequences were assigned following recommendations for a standard naming strategy^28^. All substitution models implemented in MEGA6 were tested and compared for the S2RDRP multiple sequence alignment. We selected models with the lowest Bayesian Inference Criterion score^29^ (BIC). Of the available models for pairwise distances, JTT + G had the lowest BIC. For the maximum likelihood phylogenetic trees, the model with the lowest BIC was LG + G (Supplementary Data).

### Nucleotide sequences

DRA004338 is the GenBank/EMBL/DDBJ accession number for the raw sequence reads from the metagenomics library. The nucleotide sequence for segments 1 and 2 of PBV/mouse/JPN/2015 genome were deposited in the DDBJ/EMBL/GenBank databases under accession numbers LC110352 and LC110353, respectively.

## Additional Information

**How to cite this article**: Gonzalez, G. *et al*. An optimistic protein assembly from sequence reads salvaged an uncharacterized segment of mouse picobirnavirus. *Sci. Rep.* **7**, 40447; doi: 10.1038/srep40447 (2017).

**Publisher's note:** Springer Nature remains neutral with regard to jurisdictional claims in published maps and institutional affiliations.

## Supplementary Material

Supplementary Dataset 1

## Figures and Tables

**Table 1:** Primers designed to sequence PBV/mouse/JPN/2015.

No.	Sequence	Direction	Segment	Start-End
1	TCGAACCGACACAATCTGACA*	+	S1	617–637
2	TCAGCGTTATACACTGGACC	−	S1	1732–1751
3	ACAACACCAAAGTAAGGGAAAGT*	+	S1	208–226
4	AGTCGGTGTTTTAGATGGTGTAG	−	S1	2054–2076
5	GTTTATGACACGAAACCAAATAGCGT	+	S1	1–26
6	TTAGGCGTTCAACTTCATCCTG	−	S1	392–413
7	ACATTCCGACACAGCCTACACCTGAA	+	S1	1940–1965
8	ACCCAACCAAGGTTTACGCT	+	S2	1–20
9	ATGTGGGATATCTAAACCAAGTCT	−	S2	1386–1409

^*^Primer sequence was not completely identical to the target region of PBV/mouse/JPN/2015 virus genome.

**Table 2:** Pairwise amino acid identity of PBV/mouse/JPN/2015 to other diverged PBVs.

Diverged PBVs	Accession no.	s1nsp		s1cp	
		Length (aa)	Identity (%)	Length (aa)	Identity (%)
PBV/Mouse/JPN/2015*	LC110352	241	100	577	100
PBV/pig/ITA/2004	KF861770	199	15.1	545	18.5
PBV/horse/USA/2012	KR902504	151	15.6	536	16.2
PBV/human/THA	NC_007026	224	18.7	552	18.8
PBV/human/USA/2013	KJ663813	116	15.2	552	15.2
PBV/human/NLD/2007	GU968923	213	21.6	243	8.3
PBV/turkey/USA/2011	KJ495689	252	21.8	550	20
PBV/otarine/HKG/2008	JQ776551	162	19.1	575	20.7
PBV/pig/ITA/2004	KF861768	178	16.1	615	19
PBV/fox/NLD/2012	KC692367	201	20.6	506	16.9
PBV/horse/USA/2012	KR902506	222	20.4	527	22.4
PBV/horse/USA/2012	KR902508	251	10.2	557	21
PBV/human/NLD/2008	KJ206568	129	8.4	514	19.2
PBV/dromedary/ARE/2013	AIY31265	N.D.+	N.D.+	516	20.2
PBV/dromedary/ARE/2013	AIY31272	N.D.+	N.D.+	496	20.3
PBV/rabbit/GBR/	CAB65394	N.D.+	N.D.+	590	17.4
PBV/dromedary/ARE/2013	AIY31283	N.D.+	N.D.+	465	15.6

^*^Compared to itself to provide context of proteins length.

^+^Not determined.
